# Validating gingival surface temperature as an alternative tool in the diagnosis of periodontal disease activity: An observational clinical trial

**DOI:** 10.15171/joddd.2019.019

**Published:** 2019-08-14

**Authors:** Sumanth Gunupati, Hasya Sappiti, Sreenivas Nagarakanti, BV Ramesh Reddy, Vijay Kumar Chava

**Affiliations:** ^1^ Department of Periodontology, Narayana Dental College & Hospital, Nellore, Andhra Pradesh, India

**Keywords:** Diagnosis, gingiva, inflammation, periodontitis, periodontal diseases, temperature

## Abstract

***Background.*** Elevated temperature has been recognized as an inflammatory sign. It is the only indication that can be both objectively and quantitatively evaluated and is considered as a potential indicator of periodontal disease. Assessing gingival surface temperature (GST) could be a diagnostic parameter to determine periodontal health. This pilot clinical study aimed to validate gingival surface temperature (GST) as a clinical diagnostic tool to measure periodontal disease activity by correlating with the periodontal inflamed surface area (PISA).

***Methods.*** A cross-sectional mono-center pilot study was conducted with a convenient sample of 50 participants with a mean age of 34.14±13.7 years. Clinical parameters such as probing pocket depth (PPD) clinical attachment loss (CAL) and bleeding on probing (BOP) were measured. GST was recorded with a single lead of the bedside patient monitor and correlated with PISA.

***Results.*** The results showed a positive correlation between PISA and GST (P=0.46).

***Conclusion.*** This study showed a rise in GST of inflamed sites, but the results did not support the hypothesis that increased GST is an indicator of periodontal disease. As this is a pilot study, further studies with more larger sample sizes need to be undertaken to confirm its use as a diagnostic tool in clinical trials.

## Introduction

Periodontitis is a chronic inflammatory disease of the supporting tissues around the teeth. Severe generalized periodontitis affects 5‒15% of any population worldwide and is a major cause of tooth loss and claimed to be a risk factor for a broad range of diseases such as cardiovascular diseases, stroke, preterm low birth weight and diabetes.^1^

Various methods have been used globally for clinical assessment of the disease activity, inflammation being one of the parameters. This inflammatory activity is accompanied by a sequence of events related to the release and action of an array of mediators at the inflammation site, which increase vascular permeability.^2^ The increased fluid transport in the local inflammatory region results in increased temperature. Among the five cardinal signs of inflammation, an elevated temperature is the only sign that can be both objectively and quantitatively evaluated.^3^

Elevated systemic temperature is used as a measure of disease activity in other areas of healthcare. Correspondingly, the surface temperature rises locally due to elevated blood flow in the phase of tissue destruction around an inflamed area in the skin.^4^ Furthermore, there could also be a local rise in temperature on the inflamed gingival surface.

The amount of the inflamed periodontal tissues can be quantified using periodontal inflamed surface area (PISA), as the surface area of bleeding pocket epithelium in square millimeters; it is thought to be the best tool available for quantifying the inflammatory burden posed by periodontitis.^5^ This can be calculated retrospectively from the existing research data on CAL, recession and BOP measurements.^1^

Increased temperature has been recognized as a cardinal sign of inflammation since the second century AD.^6^ Temperature is one of the methods of evaluating this response as it lends itself to direct physical measurement. Various devices have been designed to measure the temperatures of the gingival sulci, sublingual area, masticatory mucosa, labial and lingual vestibular mucosa and over peri-implant surfaces.^7^

Various studies have used customized devices for measuring the subgingival temperature, and few have used standardized Periotemp.^4,8-10^ Pioneers in this field of research stated that sulci around posterior teeth are warmer than anterior teeth.^11^ Few authors observed a higher temperature in the interdental area with the presence of plaque and bleeding on probing, suggesting that subgingival temperature rise directly reflects gingival inflammatory state.^12^

Researchers in this field also observed that mandibular gingival sulci is warmer than that in the maxilla,^6^ with some reporting no correlation between the depth of the pocket and temperature and active and inactive disease.^7,12^ To overcome the few inherent drawbacks of subgingival temperature, we measured gingival surface temperature (GST) and correlated it with PISA.

This study tested the hypothesis that gingival surface temperature can be used as a clinical diagnostic parameter for assessing periodontal disease activity by correlating it with PISA.

## Methods

A non-randomized, observational, active controlled trial was conducted in a single center following the Helsinki declaration of 1975, revised in 2013.

### Selection of Participants

The participants in this study were recruited from the Department of Periodontology, Narayana Dental College and Hospital, Nellore, Andhra Pradesh, India. A convenient sample of 50 participants was recruited for this study based on the inclusion and exclusion criteria.

The study subjects were informed about the study, and written consent was taken. The protocol of this study was obtained from the Institutional Ethics Board of Narayana Dental College and Hospital, Nellore, Andhra Pradesh, India (NDC/IECC/PER/SS/04-18/01). The trial was registered with Clinical Trials Registry-India (CTRI/2018/05/014169).

Individuals with any of the following were not included in the study: 1) use of antibiotics in the past six months; 2) pregnant and lactating women; 3) subjects with a history of any periodontal therapy in the past six months; and 4) patients with systemic diseases.

Participants aged 20‒60 years and having a minimum of 20 natural teeth were recruited; this was proposed since current inflammatory burden imposed by periodontitis requires the presence of at least a minimum number of teeth affected by periodontitis and clinical parameters of probing pocket depth (PPD) clinical attachment loss (CAL) and bleeding on probing (BOP) should be used to establish periodontitis.

### Periodontal Examination

All the participants underwent periodontal examinations, including PPD, CAL and BOP by a trained and calibrated examiner with an intra-examiner variability of 0.8. All the measurements were performed on fully erupted teeth at six sites per tooth using Williams periodontal probe (Hufriedy, USA).

CAL was defined as the distance from the cementoenamel junction to the bottom of the pocket/sulcus and calculated as the mathematical sum of the PD and gingival recession measurements;^13^ measurements were made in millimeters. The number of bleeding sites was recorded, as either present or absent within 30 seconds of probing at six sites per tooth.

PISA and periodontal epithelial surface area (PESA) were calculated by filling the spreadsheets (freely available from www.parsprototo.info.) described by Hujoel et al.^14^

### Measuring Gingival Surface Temperature

**Figure 1:**
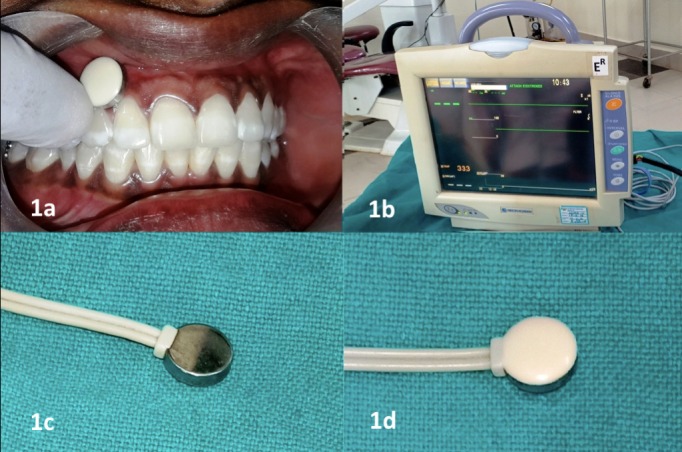
Measuring gingival surface temperature. 1a: Placement of the lead over the gingival surface. 1b: Monitor displaying the temperature. 1c, 1d: The lead used to measure the temperature.

Gingival surface temperature (GST) was recorded using a single lead of the bedside patient monitor (PVM-2701, NIKOH KOHDEN, Tokyo, Japan) which is calibrated to record temperature over the gingival surface (Figure 1). GST was recorded by a single trained examiner for all the teeth by placing the lead on both the buccal/labial and palatal/lingual gingival surfaces and waiting for 30 seconds until the temperature display over the monitor was stabilized.

### Statistical Analysis

Because the present study was a pilot study, no sample size was determined. Statistical analyses were performed using SPSS 22 (Armonk, NY: IBM Corp). Spearman’s correlation test was used to compare PISA, PESA and GST. Multiple linear regression analysis was performed for all the parameters with dependent variable as PISA. Receiver operating characteristics (ROC) curve was used to assess sensitivity and specificity.

## Results

The study comprised of 50 participants (32 males and 18 females) with a mean age of 34.14±13.7 years (Table 1).

**Table 1:** Demographic data

**Age (years)** **Mean ± SD**	**Sex**	
	**Male**	**Female**
34.14±13.7	32	18

Comparisons between PISA, PESA and GST were made by applying Spearman’s correlation test as shown in Table 2. There was a positive correlation between PISA and GST (P=0.46) and between PESA and GST (P=0.84), which were not statistically significant.

**Table 2:** Correlation between PISA, PESA, GST

		**PISA**	**PESA**	**GST**
**PISA**	**Correlation coefficient**	1.00	0.91	0.11
	**P-value**		<0.001	0.46 (NS)
**PESA**	**Correlation coefficient**		1.00	0.03
	**P-value**			0.84(NS)

Spearman’s correlation test *P<0.05, statistically significant P>0.05, non-significant (NS)

Multiple linear regression analysis was carried out for all the parameters with PISA as the dependent variable (Table 3), which showed no significant association between GST and PISA.

**Table 3:** Multiple linear regression

	**Unstandardized Coefficients**		**P-value**	**95.0% confidence interval for B**	
	**B**	**Std. Error**		**Lower bound**	**Upper bound**
**(Constant)**	-8553.85	5023.1	0.10 (NS)	-18677.24	1569.54
**Age**	0.70	5.09	0.89 (NS)	-9.56	10.96
**Sex**	175.99	90.30	0.06 (NS)	-6.00	357.99
**Tobacco**	-24.1	121.65	0.84 (NS)	-269.27	221.07
**GST**	203.16	137.76	0.15 (NS)	-74.49	480.8
**PESA**	1.15	0.06	<0.001*	1.02	1.28

Dependent variable: PISA ANOVA, F(_5,44_)=119.02, P<0.001* R^2^=0.97 *P<0.05 statistically significant P>0.05 Non-significant, (NS) Each subject was evaluated concerning PPD, CAL and the number of bleeding sites; PISA and PESA were calculated by filling the spreadsheets (freely available from www.parsprototo.info.) described by Hujoel et al.^14^

**Figure 2:**
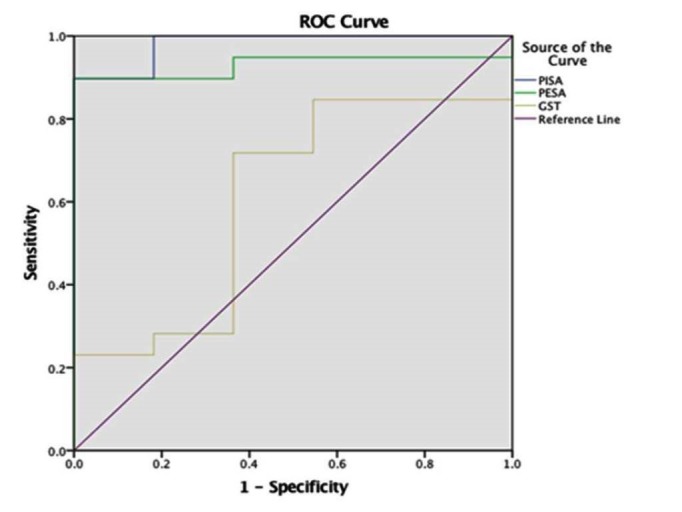
Receiver operating characteristics (ROC) curve.

To validate the accuracy of the GST compared with PISA in a better way, receiver operating characteristics (ROC) curve was used to assess sensitivity and specificity. The ROC curve showed PISA on the left top corner, representing it as a more accurate tool than GST (Figure 2). The sensitivity analysis showed a cut-off value of 35.98 for GST as a diagnostic tool (Table 4).

**Table 4:** Sensitivity and specificity of PISA, PESA and GST

	**Cut-off value**	** Sensitivity **	**Specificity**	**Area**	**Std. Error**	**P-value**	**Asymptotic 95% Confidence Interval**	
							** Lower Bound **	**Upper Bound**
PISA	509.8	0.90	1.00	0.98	0.02	<0.001*	0.95	1.00
PESA	1294.985	0.90	1.00	0.93	0.04	<0.001*	0.86	1.00
GST	35.9865	0.72	0.64	0.61	0.10	0.28 (NS)	0.42	0.80

*P<0.05, statistically significant P>0.05, non-significant (NS)

## Discussion

Many factors contribute to changes in the oral temperature.^12^ A local rise in gingival temperature has been attributed to an increase in local blood supply and tissue metabolism;^15^ clinically, these two factors cannot be assessed separately.

Changes in the color of gingiva and bleeding on probing were used to diagnose changes in gingival inflammatory status clinically. There is a subjective component in the quantitative determination of the parameters since the amount has to be estimated.^12^

Elevated temperature in a diseased state is a natural consequence of inflammatory activity. The inflammatory process is the synthesis and release of interleukin-1 (IL-1) from monocytes and macrophages. IL-1 is considered as a neutrophil pyrogen, which affects the thermoregulatory center in the brain.^16,17^ In addition, the presence of IL-1 locally contributes to increased cellular infiltration at the inflammatory sites, leading to an increase in the fluid permeation, and elevated levels of neutrophils; the consequence of these activities is a rise in local temperature.^18^

Our study aimed to provide detailed gingival surface temperature characteristics of the healthy and diseased periodontium and to determine whether site temperature differences existed between diseased and healthy periodontium. To the best of our knowledge, this is the first study measuring GST and comparing it with PISA index. The healthy and diseased sites were classified by PISA and PESA. In our study, we compared GST to PISA as it expresses gingivitis and periodontitis as a continuous variable that is a measure of the amount of the inflamed periodontal tissue.

The reason for comparing GST with PISA is that although PISA has a few shortcomings, theoretically it appears to be a better classification of periodontitis as a risk factor for other diseases than any classification currently used.

The results of the gingival surface temperature compared to PISA and PESA showed a difference between diseased and healthy subjects, and also between diseased and healthy teeth in diseased subjects; however, the differences were not statistically significant. The difference in temperature between the diseased and healthy subjects were mostly not statistically significant; as the study was designed as a pilot study, no power analysis could be performed due to the novelty of this diagnostic method. Temperature differences between anterior and posterior regions and between mandibular and maxillary arches were clinically observed; but the differences were not statistically significant.

In this study, we found the GST of mandibular teeth was higher compared to maxillary teeth, which could be plausible because the temperature within maxillary sinuses could be lower than the core temperature of the body and thus vessels running through the sinuses before supplying the maxillary teeth and gingiva could carry cooler blood.^11^ The higher temperature of the mandibular gingiva could be because the lingual artery within the mandible remains enclosed and loses no warmth before supplying the mandibular teeth and gingiva.^11^ Another reason could be the intimate contact of a highly vascularized tongue with lower jaw, contributing to less heat loss.^19^

There was also a rise in temperature of mandibular posterior gingiva than anteriors, which could be because of the natural consequence of the cooling of blood as it traverses along the arteries from the posterior region to the anterior region.^19^

There is a rise in temperature with an increase in PISA, but the results were not statistically significant; this could be because we compared GST with PISA, where PISA cannot determine the present state of disease activity. The patients we examined might have had greater probing depth and attachment loss but less inflammation.

Within the limitations of the present study, our results could not support the hypothesis that increased GST is an indicator of periodontal disease. Further longitudinal studies with larger sample sizes are necessary to validate the use of GST as a diagnostic parameter in clinical trials; moreover, comparisons should be made before and after treatment.

## Authors’ Contributions

All the authors have contributed to the critical revision of the manuscript and approved the final paper. SG, HS and SN were responsible for the concept, design, experiment and data analysis. BVRR and VKC were responsible for the literature search, drafting and proof reading.

## Acknowledgments

We would like to acknowledge Dr. Deepthi Athuluru M.D.S (Public Health Dentistry) for her contribution to the study and the participants for their kind cooperation.

## Funding

The study was self-funded.

## Competing Interests

The authors deny any conflict of interests with regard to the authorship and/or publication of this article.

## Ethics Approval

The protocol for this study was obtained from the Institutional Ethics Board of Narayana Dental College and Hospital, Nellore, Andhra Pradesh, India (NDC/IECC/PER/SS/04-18/01). The trial was registered with Clinical Trials Registry-India (CTRI/2018/05/014169).
